# SLM Manufacturing Redesign of Cooling Inserts for High Production Steel Moulds and Benchmarking with Other Industrial Additive Manufacturing Strategies

**DOI:** 10.3390/ma13214843

**Published:** 2020-10-29

**Authors:** Joaquim Minguella-Canela, Sergio Morales Planas, María Antonia De los Santos-López

**Affiliations:** 1 Centre CIM (Computer Integrated Manufacturing), Departament d’Enginyeria Mecànica, Universitat Politècnica de Catalunya, Av. Diagonal, 647, 08028 Barcelona, Spain; tania.santos@upc.edu; 2 Fluidra S.A., C/Ametllers, 6, Polinyà, 08213 Barcelona, Spain; smorales@fluidra.com

**Keywords:** additive manufacturing, selective laser melting, steel, cooling inserts, conformal cooling, moulds, multi jet fusion

## Abstract

Moulding technologies are remarkably effective for parts requiring high production volumes. Yet cooling the moulds after each injection can cause a significant loss of time. A possibility for reducing the cooling times is to use cooling inserts and conformal cooling strategies. In the present case, the original inserts of a mould must be substituted because the original material cannot be utilized anymore (toxicity). Will it be technically feasible to achieve a proper cooling only by modifying the inserts? Here, the cooling inserts of high production steel moulds utilized to manufacture ribs for swimming pool sinks’ plastic cages are redesigned, simulated and manufactured, taking advantage of Selective Laser Melting possibilities and without modifying the geometry of the obtained parts, nor the rest of the moulds. The results reveal a reduction in the mould cooling times of up to 8%, while maintaining the same conformation properties, thus leading to important savings of time and some global costs in the production outcomes. The study also benchmarks the production economic limits of this approach compared to other possible strategies, such as the development of full new conformal cooling moulds or the industrial production of the parts with plastic additive manufacturing (multi jet fusion) technology.

## 1. Introduction

Injection moulding (IM) technologies can achieve high productivity levels and seem to be one of the best technologies for producing plastic repetitive parts in high volumes. The filling of a mould can be extremely fast, and many parts can be obtained from the same injection. However, to quickly cool the very massive geometries of injected parts can be a challenging issue. In some cases, the cooling can be the most time-consuming phase of injection moulding operations [1], and if improperly carried out can induce many defects on the injected parts [2]. One of the solutions that is being used to mitigate the heat effects on the moulded parts is to use cooling inserts, which can achieve a better local cooling, thus leading to a reduction in the injection moulding cycle times.

The utilization of cooling inserts can yield better cooling results if the inserts include cooling channels [3]. The development of conformal cooling strategies [4,5] has become an industrial trend over the last decade [6,7,8]. Conformal cooling can be applied to inserts but also to full mould shapes. In [6], conformal cooling inserts were designed and produced with a ProX^®^ DMP 200 metal 3D printer (3D Systems, Inc., Rock Hill, SC, USA) on LaserForm^®^ 17-4PH (B) stainless steel material (3D Systems, Inc, Rock Hill, SC). In [8], full mould geometries were obtained in EOS Maraging Steel MSI. This technology implementation consists of implementing channels that physically follow the contours of the part to be injected. Conformal cooling strategies are used to avoid the previous cooling approaches whereby cooling channels had to be machined and/or deep drilled, thus avoiding complexity and the potentially uneven interconnection of the cooling pipes [9]. Furthermore, the use of such channels has proven to improve injection effects, such as warping and sink marks [10], and has been assessed in applications of micro injection moulding [11]. Concerning the specific industrial results in [6], it achieved a reduction of 28% in the cooling time (from 10.5 s to 7.5 s), leading to an overall reduction in IM times of 22%. In general cases [8], the reductions claimed in total cycle times can vary between 15% and a 60%, depending on part complexity.

Concerning simulation, a number of attempts have been made to determine the effects of different cooling channel designs and material utilizations on the cooling efficiency of different moulds. Zink et al. [1] defined and adjusted an enhanced simulation model considering three materials and three improvement strategies for the cooling inserts, namely conventional deep drilling, milling, and direct metal laser sintering (DMLS) manufacturing. The DMLS strategy demonstrated better results than the other two. Furthermore, the importance of the material used in the cooling inserts was demonstrated, the copper-based highly alloyed materials being the ones that exhibited a superior performance to the other options. Indeed, the use of copper-based alloys has been investigated [12]. For some AM technologies, such as selective laser melting (SLM), the process demonstrates complexity [13,14]. Furthermore, some copper-based alloys including beryllium have been banned from utilization due to the toxicity of their particles to humans [15].

Regarding the properties obtained by conformal cooling channels in injection moulds, Jahan et al. [16] conducted a design of experiments (DOE) study in order to address the objective functions of minimizing the cooling times and minimizing the maximum von Mises stresses encountered in the injected parts. Three different DOEs were conducted utilizing several cylindrical and conical plastic parts to be injected, and the effect of the cross section proved important in the design of the channels, meaning that not a single cross-section geometry provided the best results overall. Other studies addressing the design of cooling channels studied the optimal layout of piping for specific applications, such as manufacturing injected optical lenses [17]. It is accepted that cycle times can be diminished by finding the best location of gates and temperature distribution of cooling channels [18]. Furthermore, other authors [19] further developed the procedures with the obtention of automatic algorithms that allowed the automatic validation and dimensioning of the system without requiring specific user experience in mould design or simulation. Still, metal additive manufacturing (AM) technologies such as laser metal deposition (LMD) have been reported to yield potentially remarkable results for manufacturing other sorts of moulds, such as those used in hot stamping die forming [20], as well as laser-based powder bed fusion (LPBF) for stamping tools and dies, and injection moulding core inserts [21].

From the fluid mechanics point of view, another important matter when utilizing cooling channels in a mould is to make sure that the flow inside the cooling pipes follows a turbulent circulation, which can be ensured by controlling its Reynolds coefficient [22]. SLM parts are difficult to surface-finish on their interior faces, so the inside channels are expected to have a rough finish. Regardless, this feature is positive for the setting of cooling channels, as interior rugosity will help to achieve turbulence in the coolant flow. Apart from that, the outer surfaces of the SLM parts can be easily finished and heat-treated, in such a way that a smooth surface (even mirror-like [23]) and good mechanical properties could be achieved for the manufactured insert objects of the present study. AM, therefore, not only offers a new way to obtain the cooling channels, but also unleashes a design revolution for the cooling requirements of the mould industry [24]

Moving a step forward, apart from ensuring the technical viability of the solution developed, there is a need to assess whether the solution is economically viable, and to quantify to what extent, compared to other industrial approaches. On the one hand, the benefits claimed by SLM machine manufacturers [6,8,22] as regards the application of conformal cooling approaches include improved cycle times, better part quality and lower total costs of ownership. In cases wherein the insert costs are low, with the strategy conducted, the cost reductions can be seen even at low production volumes [25]. On the other hand, though, in other studies, the potential savings that could be achieved by switching from traditional manufacturing strategies (machining, IM) to AM techniques for producing final products have been shown. For some sectors, such as stock parts manufacturing, the switch to AM was proven to have significant effects, depending on certain factors which could be clustered (moderate production volumes, high number of different orders and relatively high product cost per unit) [26], with one of the most important consequences being that it could change the business models [27]. Understanding the cost factors of parts in different technologies [28,29] makes clear that the issue has to be addressed in a holistic manner through the entire product value chain [30,31]. The case for the metal AM used to produce tools is the claim of its having reached level 8 in manufacturing readiness [32], and it has been revealed to be the most favourable option so far. Still, with the rapidly evolving reductions in the cost per unit levels of the final AM industrial parts, there is a fundamental question concerning what the basis is for switching from one technology to another in a sustainable manner at the present time [33,34].

In this context, the present paper starts with the study, redesign and manufacturing of the mould features (eight core inserts) required for cooling an IM geometry (plastic ribs) produced at high volumes for assembling swimming pool sink cages. The research focus of this paper is, firstly, to develop a functional solution that can substitute an original feature of high production steel moulds (inserts) that cannot be used anymore (material toxicity of the inserts) whilst maintaining the initial mould design so as to obtain Polypropylene (PP) high volume parts and also reduce the production times (conformal cooling). This solution (considered as scenario 1) is evaluated in terms of technical feasibility and economic viability. Secondly, we conduct a study to quantify the limits of the real economic improvements than can be achieved in the present case study, compared with other manufacturing strategies such as developing full new moulds (scenario 2) or manufacturing parts with an industrial-grade AM plastic technology; i.e., multi jet fusion (scenario 3).

## 2. Materials and Methods

### 2.1. Redesign and Simulation of the Cooling Inserts

#### 2.1.1. Redesign and Material Selection of Cooling Inserts

**Figure 1:**
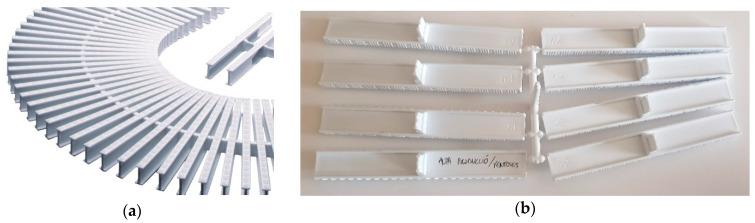
PP plastic ribs that are the object of the case study. (**a**) Assembly of a chain of ribs into a swimming pool water sink cage. The assembly can form a straight line or follow curves (picture courtesy of Fluidra group). (**b**) A set of 8 ribs resulting from an injection operation.

The mould-injected parts that are the object of the present study are Polypropylene (PP) Homopolymer ribs with external main dimensions of 195 mm in width and 35 mm in depth. The ribs can be assembled one into another to form structures used in swimming pools as water sink cages (see Figure 1a), thus obtaining the desired cage length. The plastic ribs have been manufactured in the current shape and form for many years by using injection moulding in F-442 steel moulds. Current production figures show the relatively stable demand of 13 million units per year. In the normal operation of the mould, in each injection operation, 8 of these ribs are manufactured at the same time (see Figure 1b).

**Figure 2:**
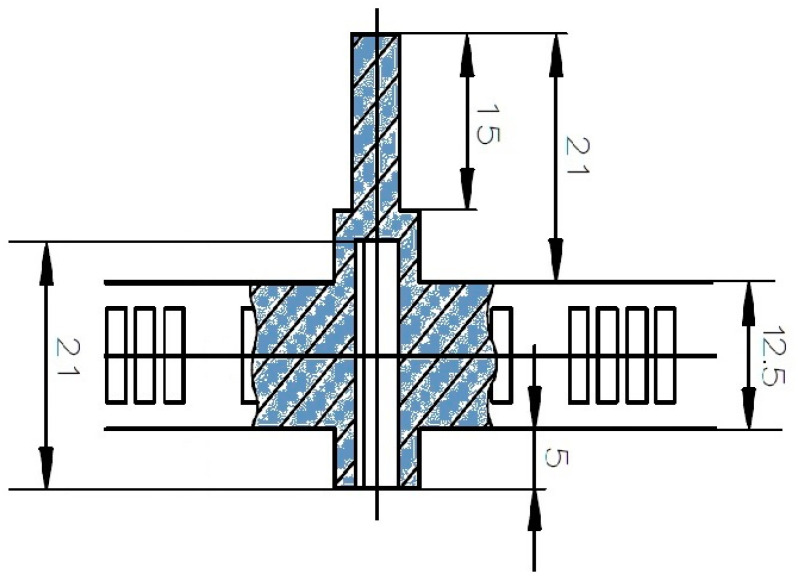
Detail of the central protrusion in a rib and its inner slot required to assemble one part into the other to form a cage (dimensions in mm).

Most of the rib geometry being a flat ‘I’ geometry of 12.5 mm height, there is a material disposition forming a central protrusion of 21 mm upwards and 5 mm downwards in a perpendicular plane to the rest of the part, giving it a maximum height of 38.5 mm (see Figure 2). This plastic material protuberance is used to assemble a rib into another one to form the cage. For this reason, on the rear part of the main material elevation, there is a slot of 21 mm of depth which is utilized to accommodate with enough clearance the axial 15 mm of the protrusion of the next rib of the cage.

**Figure 3:**
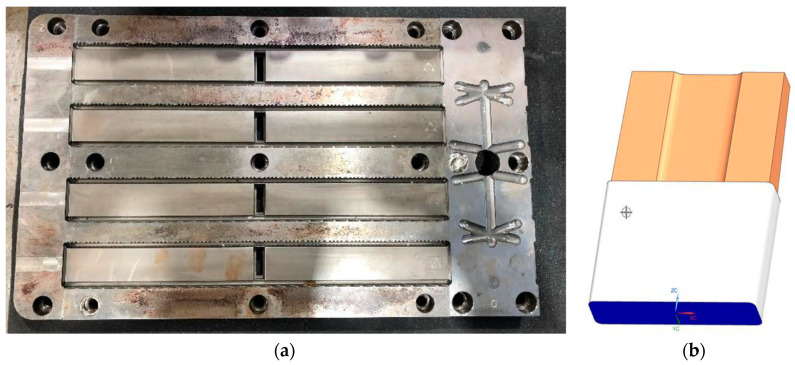
Mould disposition and key moulding elements. (**a**) Left and central sections of the ribs mould. From the depicted geometry, a total of four ribs can be extracted after a single injection operation. In each central vertical slot, one cooling insert should be placed prior to the injection. (**b**) Original cooling insert design (without cooling channels).

This geometry imposes certain difficulty on the moulding of each individual rib (see Figure 3a). Indeed, on that zone of the part, there would be a relatively high mass concentration, leading to a hot spot in the injection operation. Therefore, it requires a specific cooling enforcement to reduce the standard cooling time that would be required in the normal cycle time of the injection moulding operation. Due to its complexity and reduced size, the best solution to this problem is to use specific cooling inserts which help not only to cool down the plastic injected in the surroundings, but also to copy the shape that is required in the internal assembly slot.

The original inserts utilized in the mould (see Figure 3b) were consumables that had to be substituted after a certain number of injection moulding operations. The material of the inserts was a copper and beryllium alloy, which took advantage of the high conductive behavior of the copper and the material integrity of the beryllium. However, as mentioned in the previous section, this alloy was prescribed not to be manufacturable anymore due to the human toxicity of the compound [15].

In the search for an alternative metallic material and manufacturing technology for obtaining the cooling inserts, inox steel and additive manufacturing were respectively selected as the material (Corrax^®^ Uddeholm (Hagfors, Sweden)) and the processing technique (selective laser melting). In the selection of the material for the inserts, some other materials were also considered, such as the copper alloy Coolmould^®^, also from the manufacturer Uddeholm. Coolmould^®^ is a high-strength copper mould alloy with high thermal conductivity, which can be applied in inserts for injection moulds. However, the ribs object of the present project can also be manufactured in polyvinyl chloride (PVC), which introduces a much more abrasive behavior into the injection operation. For this reason, Corrax^®^—a precipitation hardening steel with the highest corrosion resistance (graded as 10)—is selected instead of Coolmould^®^ [35].

**Figure 4:**
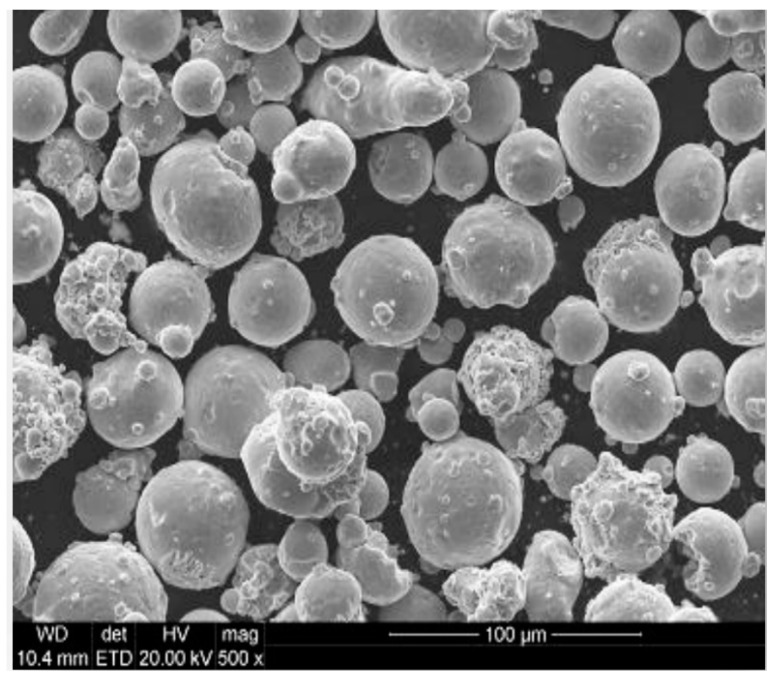
SEM investigation of AM Corrax powder raw material. Image courtesy of voestalpine group.

The shape analysis of the AM Corrax^®^ powder revealed a sphericity of 0.93 and an aspect ratio of 0.88. Figure 4 depicts the particle sizes and distribution of the powder from an SEM investigation performed with a Schottky Field Emission Scanning Electron Microscope JEOL JSM-7610F (Jeol Ltd, Tokyo, Japan).

**Figure 5:**
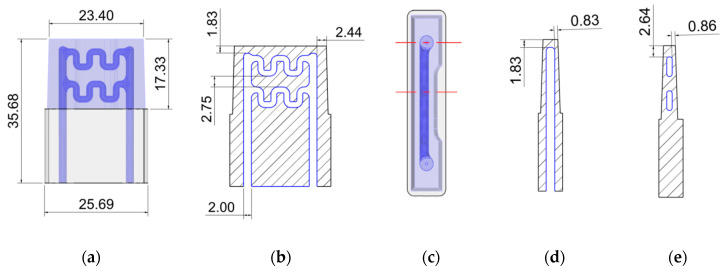
Cooling insert redesigns, incorporating cooling channels. (**a**) Exterior sizes of the cooling insert redesigns (front view). (**b**) Cooling channel dimensions of the cooling insert redesigns (front view). (**c**) Cooling insert redesigns with indication of sections (**d**) and (**e**) (top view). (**d**) Cooling channels side minimum distances to the exterior of the insert (side view). (**e**) Cooling channels central minimum distances to the exterior of the insert (side view). Dimensions in mm.

With this combined material and technology -Corrax^^®^^ and Selective Laser Melting (SLM)-, it is possible to develop a new design for the cooling inserts [21] which could include intricated internal piping cooling geometries (see Figure 5a–e). Furthermore, and concerning the material selection, it must be taken into consideration that in the cooling channels, the coolant will also have the potential to corrode the inserts. Therefore, the selection of a material with the most resistant behavior to corrosion (Corrax^®^) is again of much interest for the overall expected results.

#### 2.1.2. Simulation of the Injection Moulding Operation

The simulation of the injection moulding operation was performed with the software Moldex3D (CoreTech System Co., Ltd., Zhubei City, Taiwan). The injected material simulated was PP Homopolymer SABIC PP 575P (Songhan Plastic Technology Co., Ltd., Shanghai, China), which is the material in which the parts are manufactured for serving the demand. This material was considered to fill in an average thickness of 3.0 mm in each section of the part at a melt temperature of 230 °C while the mould was considered to be held at a temperature of 40 °C. This temperature was selected due to the material (PP) and to the geometry of the parts to be injected. Indeed, the ribs are parts with a relatively long width compared to the height and at the same time very thin; thus, being prone to deformation errors when being injected.

The cooling medium used in the simulation was water, entering the cooling circuit at a temperature of 20 °C (ejector side) and exiting the cooling circuit at a temperature of 40 °C (injector side). The chiller pressure was fixed at 2.5 bar to ensure a turbulent flow [36] inside the cooling channels, and the plastic injection time required (material filling only) was 1 s.

### 2.2. Manufacturing of the Cooling Inserts

#### 2.2.1. Additive Manufacturing of Inox Steel Inserts: Selective Laser Melting (SLM)

As mentioned in the previous section, the material used was the commercially available composition AM Corrax^®^ (Uddeholm, Hagform, Sweden), which is an inox steel specifically developed to obtain moulds via AM applications. According to the provider specifications, the material contains (wt. %) C (0.03), Si (0.3), Mn (0.3), Cr (12.0), Ni (9.2), Mo (1.4) and Al (1.6).

The manufacturing of a set of 8 inserts was performed in an SLM machine EOS M290 (EOS GmbH (Electro Optical Systems), Krailling/Munich, Germany) with a prescribed resolution of 0.05 mm. The machine has a building volume of 250 × 250 × 325 mm^3^ and its laser source is a Yb fiber laser. Concerning the process parameters, following the material manufacturer indications, the laser power was set at 170 W and the scanning speed at 1250 mm/s. The scanning strategy utilized was striped with an overlap of 10% from 0.10 mm. The hatch pattern choice was that of the default for DMLS EOS equipment (direction of scanning rotated 67° between consecutive layers). The orientation of the parts in the construction platform was vertical (in plane y–z). The layer thickness in the Z-direction was set at 30 μm. With this selection of parameters, the total build time for the completion of a platform with the parts was 20 h.

#### 2.2.2. Post-Processing Operations

The metal inserts were removed from the construction platform by performing a cross-cut operation with a saw machine Klaeger Bitron 300 3D Cut (Klaeger Sägetechnik GmbH, Kernen, Germany).

The as-printed microstructure of the inserts was nickel martensite with about 20% retained austenite. The samples were heat-treated with a solution annealing at 850 °C for 30 min and then cooled quickly to transform the retained austenite to martensite. The nickel martensite was quite soft so the hardness was about 35 HRC, but following the instructions of the material manufacturer, the produced inserts were heat-treated (ageing) for 4 h at 525 °C to achieve a hardness of 50 HRC. The cooling was performed leaving the parts in air until they reached the ambient temperature. As predicted by the material manufacturer, the ageing provoked a small and uniform contraction in all the part’s directions of 0.07%, which was already considered in the original dimensional design of the range of tolerances of the inserts.

No other surface finishing processes were performed as the outer geometry of the insert is copied on the inside notch of the ribs, which are not visible when the assembly of the cage is completed.

### 2.3. Testing and Validation

#### 2.3.1. Material Properties for Testing of the Overall Mould Performance

Having followed all the instructions from the material manufacturer, the AM Corrax^®^’s material properties considered for the numerical simulation are those presented in the materials technical sheet [37], which is reproduced in Table 1 and Table 2.

Regarding the PP Homopolymer described, the applicable material properties considered for the numerical simulation are those presented in the materials technical sheet, provided in [38] and also reproduced in Table 3.

#### 2.3.2. SLM Insert Samples

**Figure 6:**
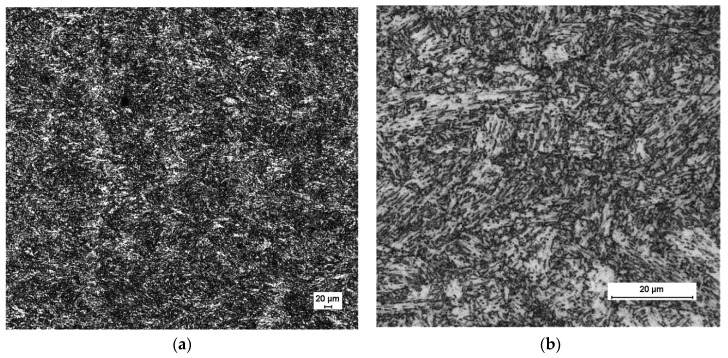
SEM investigation of the surface of an SLM manufactured part (insert). (**a**) Magnification at 50×; (**b**) magnification at 500×. Image courtesy of voestalpine group.

Having followed all the considerations given by the material provider, Figure 6 depicts an SEM investigation of the surface finish of the SLM as-built samples, performed with a Schottky Field Emission Scanning Electron Microscope JEOL JSM-7610F (Jeol Ltd, Tokyo, Japan).

The roughness characterization of an as-built part revealed a roughness parameter Rz of 25 µm, which was a parameter needed for the simulation. This value for the roughness parameter was applied to the material in the virtual model analyzed.

## 3. Results and Discussion

### 3.1. Performance of the Cooling Inserts Based on Numerical Simulation

The performance of the cooling inserts was assessed based on the analysis of the numerical simulation results conducted with the information presented in the previous section. Firstly, we analyzed the time evolution of the temperature distribution of a rib from the start of the material injection.

**Figure 7:**
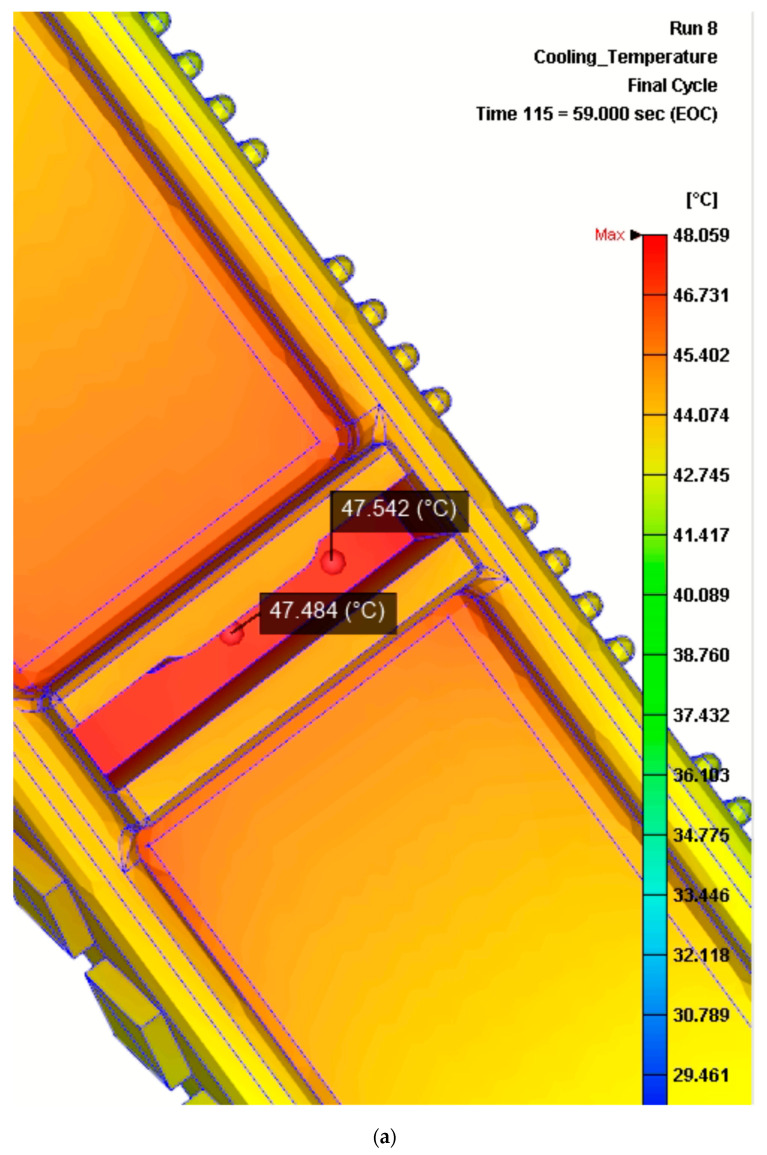

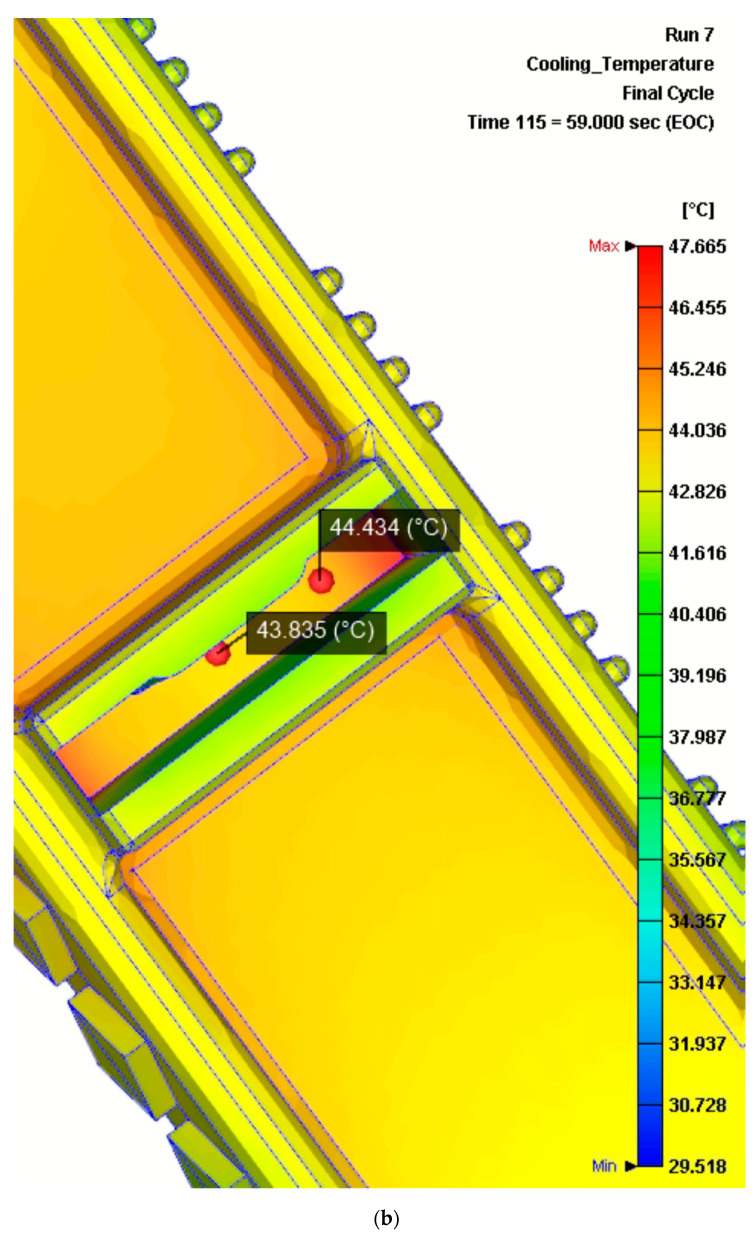
Results of the rib temperature distribution numerical analysis obtained at time = 59 s with Moldex3d. (**a**) Temperature distribution of a rib using the original insert (without conformal cooling). (**b**) Temperature distribution of a rib using the redesign of the insert (with conformal cooling channels).

The original cycle time started with the injection itself (which lasted 1 s), the holding time (lasting 8 s), and finally the cooling time, which was 50 s. This means that, using the original inserts, the reference time before opening the moulds was a total of 59 s in total. Therefore, the evolution of the temperature distribution across the time was measured until a final time of 59 s (see Figure 7). At this time, the results for the original inserts design revealed that the central hot spot of the ribs reached a final temperature of approximately 47.5 °C. Likewise, the temperature distribution analysis at 59 s, using the inserts redesigned to include conformal cooling channels, resulted in a temperature of approximately 44 °C.

These results, which proved positive, demonstrated that, despite switching the material from a more conductive option (copper-based alloy) to a less conductive option (steel), the redesign of the inserts was capable of cooling down the hotspot in an even shorter time than the original.

**Figure 8:**
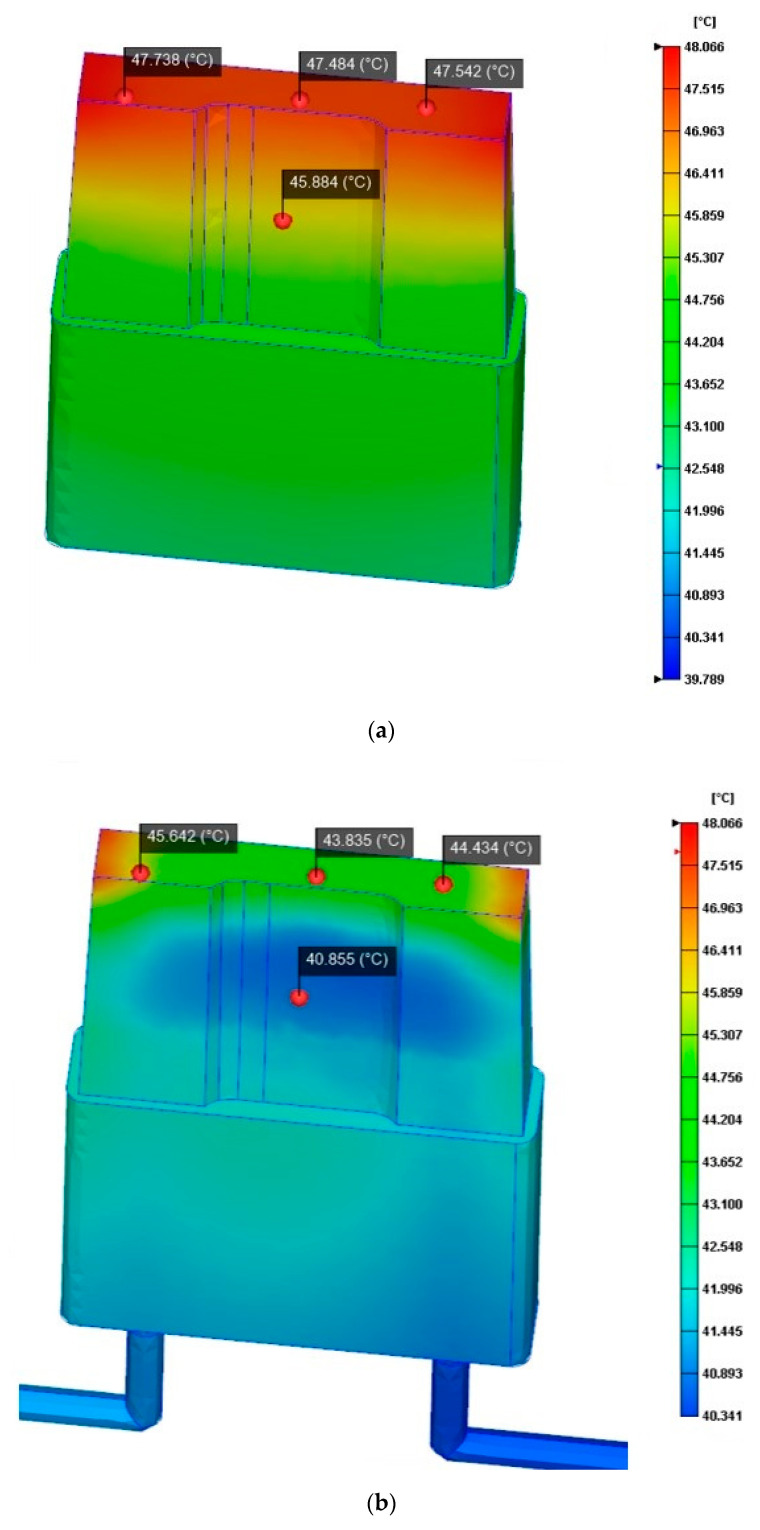
Results of the insert temperature distribution numerical analysis obtained at time = 59 s with Moldex3d. (**a**) Temperature distribution of the original insert (without conformal cooling). (**b**) Temperature distribution of the redesign of the insert (with conformal cooling channels).

Knowing that the performance of the redesign was good, we further investigated the evolution of the temperature distribution in the inserts during the cycle time (see Figure 8). As expected, the maximum temperature of the new inserts was lower than the maximum temperature reached in the original ones (approximately 45 °C compared to approximately 47 °C). In particular, the top internal zone where the cooling channels are placed revealed a heatsink effect leading to a temperature reduction of almost 5 degrees (from 45 °C to 40 °C, approximately).

**Figure 9:**
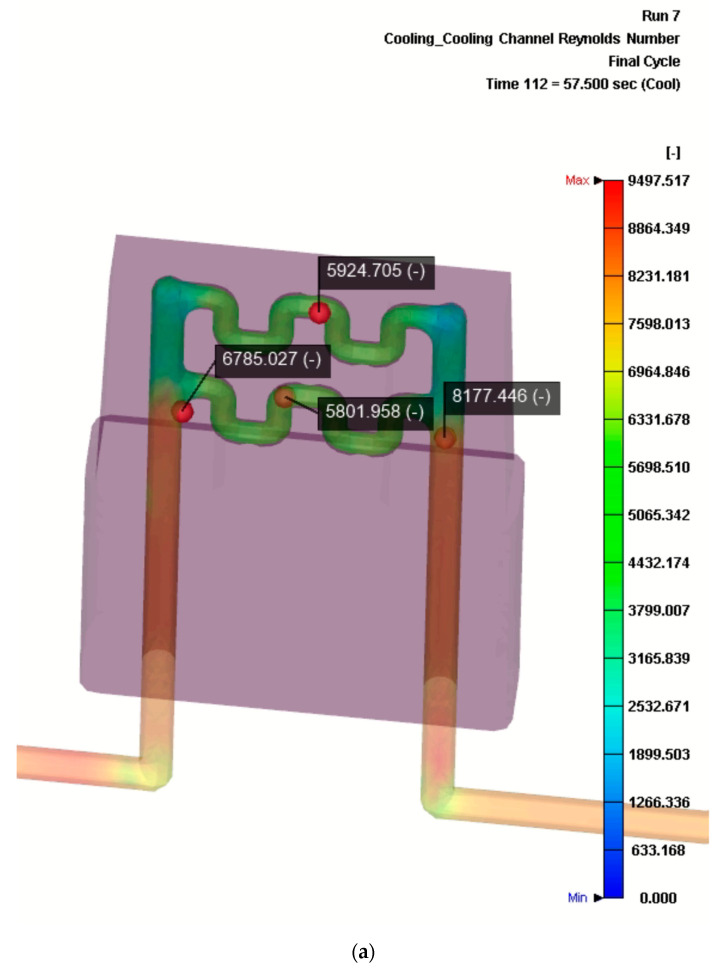

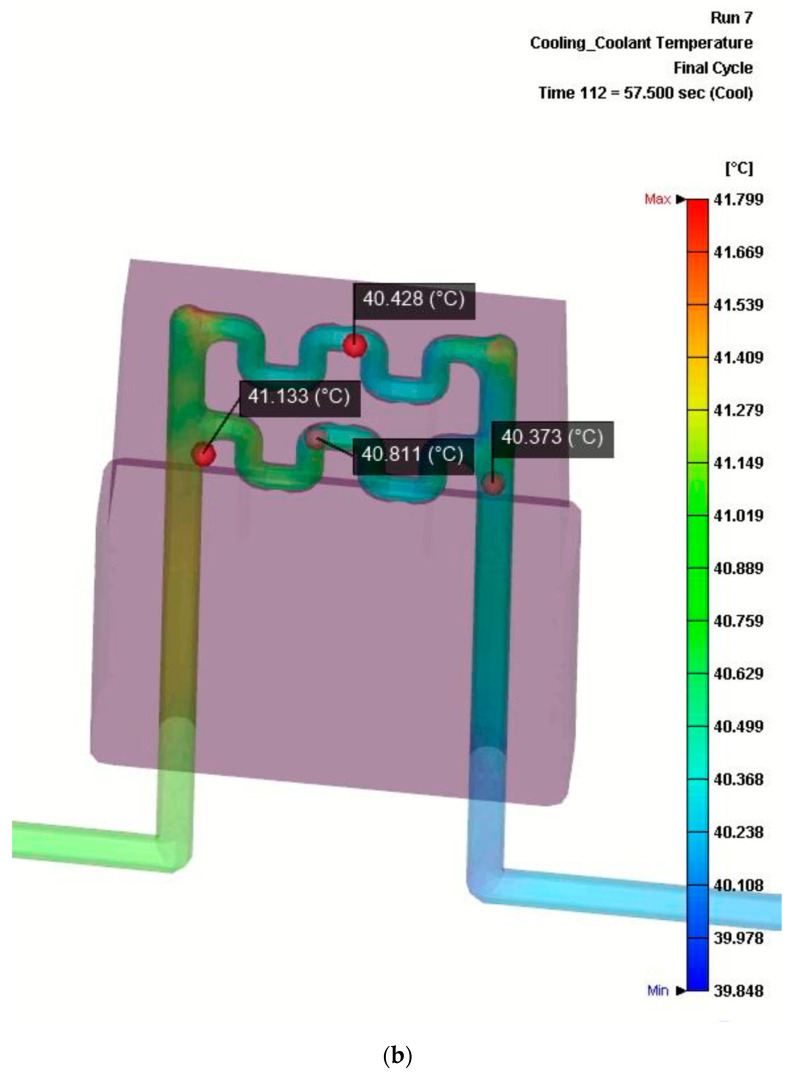
Results of the numerical simulation of the cooling flow obtained at time = 57.5 s with Moldex3d. (**a**) Cooling channel Reynolds number of the redesign of the insert (with conformal cooling channels). (**b**) Temperature distribution of cooling flow in the redesign of the insert (with conformal cooling channels).

Finally, it was interesting to evaluate the Reynold’s number distribution and the temperature distribution of the cooling fluid inside the cooling channels (see Figure 9).

On the one hand, Figure 9a demonstrates that the cooling flow inside the channels remains in the turbulent range during the simulation, taking values between 5801 and 6785 at different points of the flow. On the other hand, Figure 9b shows that the temperature flow of the cooling fluid inside the conformal channels ranges between approximately 40 °C at the entrance and 41 °C at the exit of the piping.

With these simulations, it is demonstrated that the ratios of improvement that it is possible to achieve are between −7.4 and −7.6% of the temperature decrease in both the product and the inserts, while maintaining a turbulent Reynolds condition. All these results are summarized in Table 4.

Then, taking into consideration that the cooling of the hotspot takes place faster in the design with cooling channels than in the original situation, it is interesting to find in the temperature distribution evolution at which moment the injected ribs with the new insert disposition met the original cooled temperature of 47.5 °C. This moment was when the time was 46 s, meaning that the original required cooling time (50 s) could be reduced by 4 s when utilizing the new conformal cooling inserts.

This reduction of four seconds in the cooling operation (8% of the cooling time) accounts for a reduction of 6.25% in the total injection moulding cycle time overall (see Table 5).

The reductions in the injection moulding cycle times are in close proximity to what is found in other case studies in the literature. Trying to find the edge of performance of these sorts of solutions, we can find some case studies that claim higher figures, such as reductions of up to 22% of the injection moulding cycle times [6]. However, in the present case study, the time reduction achieved is satisfactory because one of the reasons for substituting the material is that the original one cannot be used anymore due to its toxicity. Under these circumstances, the redesign objectives were to reach at least the same performance level as the original solution, which has even been exceeded. Interestingly, the material of the inserts was substituted by an option which initially was supposed to be less favorable (with lower conductivity), and yet has been revealed to be capable of improving the former system by deploying the necessary parameters.

### 3.2. Economic Comparison between the Scenarios Yield by the Original Inserts and the Inserts Redesign 

To fully evaluate the case, it is interesting to complete the production times analysis with a full economic evaluation of the implementation of the redesign, taking into consideration not only the costs of production (injection operations), but also the costs of manufacturing the inserts containing conformal cooling channels by means of SLM.

As a framework for comparison, we can use a variation of the cost schema presented in Minguella et al. [39]. In particular, the cost per produced unit can be calculated as in Equation (1):

(1) $$Ct\left[\frac{m.u.}{\mathrm{year}}\right]=Cprep+Ci+Cr$$

where:

- *Ct* is the total annual cost of manufacturing the number of annual desired units (m.u./year);
- *Cprep* is the total annual cost of the preparation of the production of batches in order to manufacture the desired number of units (m.u./year);
- *Ci* is the total annual cost of investments needed in the specific manufacturing system (m.u./year);
- *Cr* is the total annual cost of the rest of factors independent from the batch size needed in order to manufacture the number of desired parts (m.u./year).

As an important simplification of the original model in this case, stock costs are not considered, as it is supposed that all the produced parts are shipped straight after their production, and so the production facility does not hold any of these.

The two manufacturing options presented are referred as Scenario ‘0’ (using the original inserts) and ‘1’ (using the inserts redesigned with internal cooling). As in the present analysis the total annual demand (set to 13,000,000 units) cannot be met by a single machine, it is important to limit the analysis to the maximum productivity that could be reached by a single operating machine. For this matter, it is supposed that a single machine works 24 h per day, 220 days per year. These figures, considering the processing times per part obtained from the simulations above (*Tc*_0_, *Tc*_1_), enable one to calculate the maximum productivity, which is also the batch size to be used (*B*_0_, *B*_1_). Preparation times ‘*Tp*_0_’ and ‘*Tp*_1_’ are considered to be 3 (h/batch) and the cost per hour of preparation ‘*Chp*_0_’ and ‘*Chp*_1_’ are 15 (EUR/h).

The annual cost of investments needed for this comparison excludes machinery and considers only the specific tooling required. In the original case, ‘*Ci*_0_’ is EUR 4500, which equals the yearly cost of manufacturing spare inserts. In the redesign scenario, ‘*Ci*_1_’ is EUR 9600, which is the cost of manufacturing the new additive manufacturing inserts. 

Furthermore, the processing cost per part calculation (*Cproc*) considers the processing time per part (*Tc*) and the hourly running facility costs (*Ch*) as presented in Equation (2):

(2) $$Cr\left[\frac{m.u.}{year}\right]=\left(Cproc+Co\right)\times X=\left(Tc\times Ch+Co\right)\times X$$

where:

- *Cr* is the total annual cost induced by the rest of the factors independent from the batch size in order to manufacture the number of desired parts (m.u./year);
- *Cproc* is the processing cost of manufacturing a part (m.u./ut);
- *Co* is the cost of the other factors per unit independent from the batch size (materials) (m.u./ut);
- *X* is the number of processed parts, which in this case is equal to the batch size ‘B’ (ut);
- *Tc* is the processing time per part (m.u./year);
- *Ch* is the hourly running facility costs (m.u./h).

In the calculation of the hourly running facility costs of both scenarios (‘*Ch_0_*’ and ‘*Ch_1_*’), we include the amortization of the injection machine (EUR 150,000 for 7 years of 220 working days each) plus the hourly labor cost of an operator running the machine (15 EUR/h). As a frame for comparison, we decided not to incorporate the reject rates in the scenarios deliberately. The service lives of the moulds are considered to be longer than the framework for comparison.

With all these considerations, the main parameters obtained for the comparison of scenarios ‘0’ and ‘1’ are synthesized in Table 6.

The total costs calculations can be translated into costs per unit if dividing the calculated figure by the number of parts produces ‘*X*’. When doing so, the deployment of the cost functions for scenarios ‘0’ and ‘1’ has the form of a couple of curbs that cross at an equilibrium point. This equilibrium point can be calculated by equalling the two cost functions as presented in Equations (3) and (4):

(3) $$\frac{Ct_{0}}{X}=\frac{Ct_{1}}{X}\;\Leftrightarrow \;\frac{Cprep_{0}+Ci_{0}+Cr_{0}}{X}=\frac{Cprep_{1}+Ci_{1}+Cr_{1}}{X}$$

(4) $$\frac{Cprep_{0}}{X}+\frac{Ci_{0}}{X}+\left(Tc_{0}\times Ch_{0}+Co_{0}\right)=\frac{Cprep_{1}}{X}+\frac{Ci_{1}}{X}+\left(Tc_{1}\times Ch_{1}+Co_{1}\right)$$

As *Cprep*_0_ *= Cprep_1_*, the expression can be arranged as in Equations (5) and (6).

(5) $$\left(Tc_{0}\times Ch_{0}+Co_{0}\right)-\left(Tc_{1}\times Ch_{1}+Co_{1}\right)=\frac{Ci_{1}-Ci_{0}}{X}$$

(6) $$X=\frac{Ci_{1}-Ci_{0}}{\left(Tc_{0}\times Ch_{0}+Co_{0}\right)-\left(Tc_{1}\times Ch_{1}+Co_{1}\right)}$$

Substituting the values of Table 5 in Equation (6), the equilibrium figure for these two scenarios is X = 1,741,302.54 parts (below the maximum batch size per machine). This means that the scenario (1) is economically more favorable, starting at a level of 1,741,303 produced parts at a cost of 0.0995 EUR/unit and onwards at an even lower cost.

Knowing that one machine could manufacture 2,376,000 parts per year with the original inserts, the original design required six machines to meet the demand of 13,000,000 parts. If fully occupied, those machines were capable of manufacturing 14,256,000 parts per year. The reduction in processing times obtained by the cooling inserts, apart from the reduction in the unit costs in the long run, makes it possible to enlarge the production to a figure of 15,206,400 parts with the same number of machines, meaning that extra capacity could be met with the same machines (positive in cases where increases in demand could occur). Furthermore, if the demand were to diminish to at least 12,672,000 parts, scenario (1) could cover the manufacturing with only fix machines. 

Finally, in a situation where the demand is set to 13,000,000 parts per year, six machines would be producing 2,166,667 parts on average, meaning that scenario (1) would be more favorable from an economic point of view. Indeed, the 6.25% decrease in total processing time accounts for a total of 1805.6 hours to serve the overall demand of 13,000,000 parts.

### 3.3. Benchmarking with Other Industrial Additive Manufacturing Strategies

#### 3.3.1. Economic Analysis of a New Conformal Cooling Entire Mould Redesign

Having seen in the literature the massive savings made possible by the application of conformal cooling strategies in entire mould redesigns [8], the natural benchmark for the present study is to assess the results that could be achieved by undertaking a full redesign of the mould, incorporating conformal cooling, and in this way to assess the feasible performance limits in terms of both costs and processing times.

In this new a scenario, referred as (2), the processing time in each injection operation is estimated to achieve a reduction due to the application of conformal cooling aligned with what is found in the literature for parts of these sizes and geometries—namely, 20%. From the original 64 s, we envisage reaching a total time of 51.2 s. In addition, trying to maximize the output, there is the possibility of increasing the number of repetitions of the part produced in every injection operation, as the platen size of the equipment is 959 × 905 mm^2^ and the dimensions of the current mould are 900 × 500 mm^2^. With the current part dimensions, it is estimated that the mould could host up to 14 repetitions of the ribs. In this configuration, the service specifications of the equipment would also be met. The equipment used is a Topfine CE Series 2000-270 IM machine (Topfine Precision Machinery Company Ltd, Hong Kong) with a maximum locking force of 2700 kN. Working at a service pressure of 175 MPa for PP and with a projected surface of each component of approximately 301 mm^2^ = 0.000301, 14 repetitions would account for 0.004214 m^2^. The required closing pressure would be 737.45 kN (175 MPa × 0.004214 m^2^), far below the maximum locking force of the equipment.

With these considerations and in particular with the new much reduced figure for the processing time per part (*Tc_2_*) of 3.7 s, the maximum batch size to be manufactured in one year by a single machine increases to 5,197,500 units. The preparation costs (*Cprep*) decrease to EUR 30 as the moulds can be prepared more easily than in the previous scenarios. However, a relatively big investment cost must be considered, estimated at EUR 252,000, in the design and development of all the parts in the mould, which would be manufactured via SLM. Following the rules of the company, this figure is to be depreciated over a period of five years. As a summary, all the parameters for comparing the cost levels for the unit produced in scenario (2) can be found in Table 7.

Scenarios (1) and (2) (Table 1 and Table 2) are both implementations of manufacturing strategies via injection moulding technologies. For this reason, the costing model applied to the previous scenarios (0) and (1) (Table 1) can also be applied to the mould redesign strategy. The cost per unit function of scenario (2) takes again the form of a decreasing curb, with higher costs per unit at low production quantities, but with lower costs per unit once the production quantities increase.

Having found the equilibrium point between scenario (0) and scenario (1), it is of interest to find the cutting point of strategies (1) and (2) to be able to assess when it would make sense to aim for this production strategy. Applying Equation (6) to compare scenarios (1) and (2), and substituting the parameters in the equation, we found the equilibrium point at 1,812,508.2 produced parts. This means that scenario (2) is economically more favorable, starting at a level of 1,812,509 produced parts at a cost of 0.0992 EUR/unit, and onwards at even lower costs.

**Figure 10:**
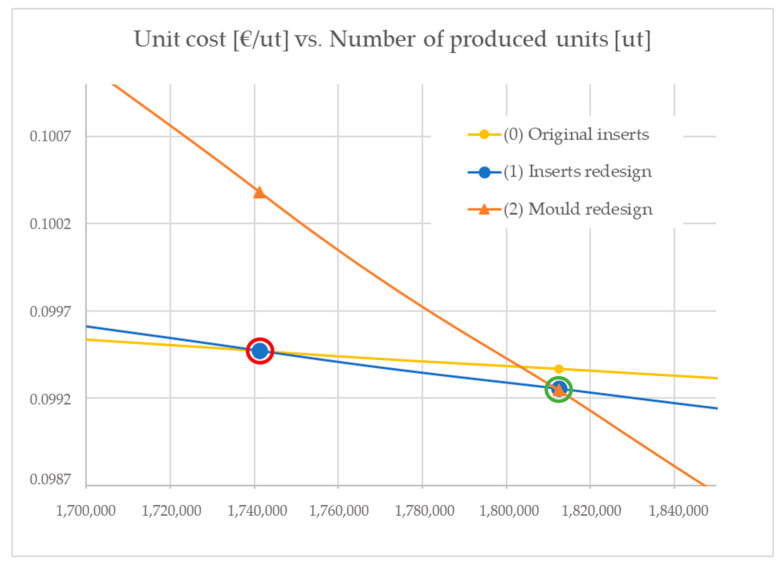
Equilibrium points when analyzing the costs per unit produced as a function of the number of produced units for the three injection moulding strategy scenarios: (0) original inserts, (1) insert redesigns and (2) mould redesigns. Equilibrium between (0) and (1) occurs at 1,741,302.5 produced parts (red circle) and equilibrium between (1) and (2) at a figure of 1,812,508.2 produced parts (green circle).

The two equilibrium points found (between (0) and (1) and between (1) and (2)) are depicted in Figure 10.

With the maximum batch size of scenario (2), it would be possible to meet the production of a figure of 31,185,000 parts with six machines, and only three would be needed to manufacture the 13,000,000 units of the annual demand. If the demand were to diminish to at least 10,395,000 parts, scenario (2) could cover the manufacturing with only two machines.

Finally, in the situation of the demand set to 13,000,000 parts per year, five machines would be producing 4,333,333 parts on average, meaning that scenario (2) would be more favorable from the point of view of the cost per unit. However, the level of savings would fall far below the 18% obtained in the literature due to the economic implications of the need to invest in the new mould development.

Regardless, the combination of conformal cooling in moulds with the maximum utilization of the IM equipment would dramatically decrease the processing time per part, thus further reducing the number of hours required to serve the overall demand.

#### 3.3.2. Economic Analysis of the Production of Swimming Pool Sink Cage Ribs via Multi Jet Fusion Technology

Scenarios (0), (1) and (2) refer to injection moulding strategies to manufacture the parts. These technologies require the preparation and use of specific tooling that implies a fixed cost independent of the number of units produced. Given the emergence of additive manufacturing industrial technologies that can compete with traditional processes in obtaining relatively long runs of production, it is of interest to compare the three initial scenarios with the results that can be achieved nowadays via direct (AM based) manufacturing.

One AM technology in particular that is progressing fast and obtaining relevant results is multi jet fusion, which is being assessed intensively concerning its process characteristics [40,41,42] and to determine the maximum capabilities that it can yield at the current time [43,44,45,46]. HP Inc. offers a new HP 3D High Reusability PP15 enabled by BASF, SE [47], which could be of use for such parts, as the mechanical property yields are the most similar.

In the present assessment (scenario (3), Table 8), we are considering manufacturing using the equipment of MJF 4210 (HP, Palo Alto, CA, USA) with a maximum effective building volume of 380 mm × 284 mm × 380 mm. With this platform’s dimensions, a total of 104 units can be manufactured in the same batch. The construction of a full platform could be undertaken using two different strategies—namely, pass print mode fast (with an expected duration of 10.5 h) and pass print mode balanced (with an expected duration of 15 h). As the objective of the present study is to explore the boundaries of this technology, the former (and faster) strategy is selected.

In this case, the hourly running facility costs (*Ch*_3_) rise significantly from the cost levels of the previous scenarios, as the machinery costs of MJF are higher than those of IM. The estimation used considers the depreciation of the equipment in 7 years, utilizing the machine 24 h/day for 220 days per year, as well as some associated labor costs.

As a significant difference between scenario (3) and the previous scenarios, MJF is a flexible process which incurs no costs of tooling. On the contrary, its preparation costs are higher, as the process requires one to prepare the different batches. The summary of the main parameters for the unit cost and time comparison can be found in Table 8.

Regarding the cost function for scenario 3, Equation (1) remains valid. However, due to the batch nature of the AM technologies, the total preparation costs per part will depend on the number of parts manufactured to meet the overall demand, in relation to the maximum number of parts in a batch, according to what it is presented in Equation (7):

(7) $$Cprep_{3}=\frac{X}{B_{3}}\times \left(Tp_{3}\times Chp_{3}\right)$$

where:

- *Tp* is the preparation time (h/batch);
- *Chp* is the preparation cost per hour (m.u./h).

Considering this and that the tooling costs (*Ci_3_*) are equal to zero, the equilibrium point between the cost levels per produced part in scenarios (0) and (3) can be obtained as presented in Equations (8) and (9).

(8) $$\frac{Ct_{0}}{X}=\frac{Ct_{3}}{X}\;\Leftrightarrow \;\frac{Cprep_{0}+Ci_{0}+Cr_{0}}{X}=\frac{Tp_{3}\times Chp_{3}}{B_{3}}+\frac{Cr_{1}}{X}$$

(9) $$X=\frac{Cprep_{0}+Ci_{0}}{\left(\frac{Tp_{3}\times Chp_{3}}{B_{3}}\right)+\left(Tc_{3}\times Ch_{3}+Co_{3}\right)-\left(Tc_{0}\times Ch_{0}+Co_{0}\right)}$$

**Figure 11:**
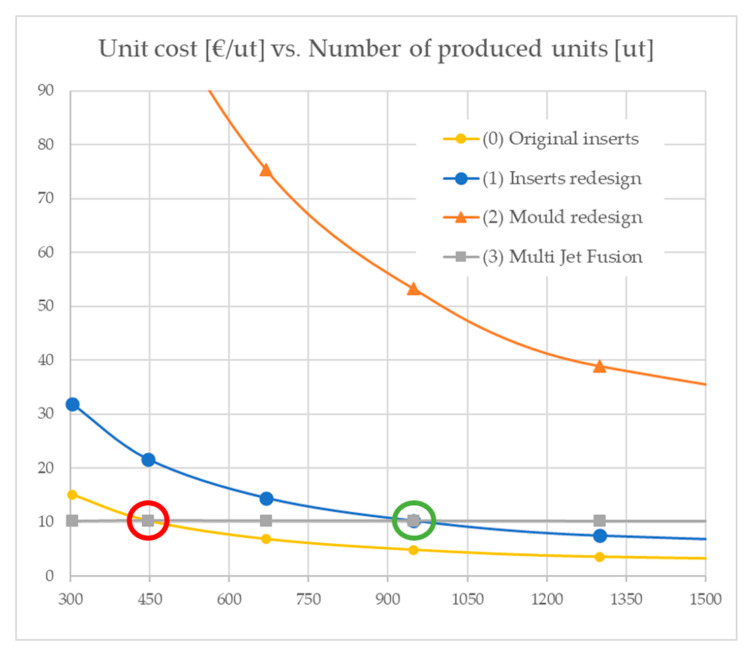
Analysis of the costs per unit produced as a function of the number of produced units for the four scenarios: (0) original inserts, (1) insert redesigns, (2) mould redesigns and (3) multi jet fusion. Equilibrium between (0) and (3) occurs at 446.9 produced parts (red circle) and equilibrium between (1) and (3) at 947.4 (green circle).

Substituting the values of the scenarios (0) and (3) (Table 5 and Table 7) in Equation (9), the equilibrium figure for these two scenarios is shown to be X = 446.9 parts (far below the maximum batch size for an injection machine in any of the scenarios evaluated) at a cost level of over 10.2646 EUR/part. The equilibrium point found between (0) and (3) is depicted in Figure 11, which also describes the cost levels at low production runs for all the explored scenarios.

The results obtained in scenario (3) yield, as expected, levels of cost per part which are still far from those that can be obtained via injection moulding in a medium long series. However, it is very noticeable that the cost per part achieves the point of equilibrium after several hundred produced parts, which is a lot considering what it was just in the recent past. Additionally, as scenario (0) will not be feasible anymore due to the toxicity of the material utilized in the inserts, it is remarkable that the equilibrium point between (3) and (1) reaches a figure of 947.4 produced parts, with a cost of 10.2741 EUR/part. 

Furthermore, given the production times and the batch size, a single machine could manufacture a total of 52,297 units of swimming pool sink cage ribs per year, without needing to develop specific moulds.

The cost per part is expected to decrease in the near future as the equipment cost and especially the material costs decrease. In any case, apart from the short series cases and the help in some production peaks, MJF remains a very interesting technology to achieve the manufacturing of complex designs that could not be achieved via injection moulding.

## 4. Conclusions

The study succeeded in finding a substitute for an original feature of the high production steel moulds (inserts) that cannot be used anymore (material toxicity of the inserts), whilst maintaining the initial mould design for obtaining PP high-volume parts, and being timely and economically favorable.

The manufacture of the inserts via SLM on Corrax^®^ made possible the application of conformal cooling AM strategies. Information reported from the SLM printed parts was utilized in the simulation analysis and for validating the manufacturability of such redesigns. The analysis by means of simulation revealed that the cooling channels decrease the injection material temperature distribution sufficiently to demonstrate the feasibility of the reductions in the original cooling time and processing time overall. Regarding cost levels, the insert redesign strategy proved to be favorable within the annual demand figures. The cost reduction obtained in the present study is not higher than results claimed in the literature. The implementation is considered successful as it meets the requirements of lowering the processing times and substituting the original inserts’ material.

The scenario analysis for developing entire new conformal cooling moulds proved to be economically favorable, starting at a number of manufactured units higher than in the inserts redesign strategy but lower than the number of manufactured units per year. Regardless, the development of an entirely new mould entails a large investment, which would have to be addressed very carefully by the company.

Finally, in the test with the application of the direct manufacturing of the parts with plastic AM (MJF), although the costs per part are relatively high for meeting the annual demand, the result advocates that the introduction of changes in the value chain can be of major importance for cost-saving in the overall operations of the company, especially when considering that the present study does not aim to study the costs of associated operations, such as stock or order shipment and distribution across the globe.

As the production levels in this case reach values of millions of units, the most favorable scheme is IM, with savings achieved by the cost- and time-reductions yielded by the cooling times (the first target of the study). The findings and methodologies of this study will be explored for other products with similar characteristics.

## Figures and Tables

**Table 1:** Mechanical characteristics of AM Corrax^®^ material (I).

Mechanical Characteristics of AM Corrax^®^ Material (I)	Values at 20 °C	Values at 200 °C	Values at 400 °C
Density (kg/m^3^)	7700	-	-
Elasticity module (N/mm^2^)	200,000	190,000	170,000
Thermal expansion coefficient calculated from the dimensions at 20 °C (%)	-	11.7 × 10^−6^	12.3 × 10^−6^
Thermal conductivity (W/m °C)	1800		

**Table 2:** Mechanical characteristics of AM Corrax^®^ material (and II).

Mechanical Characteristics of AM Corrax^®^ Material (II)	Values for 50 HRC
Elasticity Limit Rp 0.2 (MPa)	1600
Maximum traction strength (MPa)	1700
Maximum yield at 5 (%)	10
Maximum compression strength (MPa)	1800

**Table 3:** Mechanical characteristics of PP Homopolymer SABIC PP 575P.

Mechanical Characteristics of PP Homopolymer SABIC	Values at 20 °C
Density (kg/m^3^)	905
Maximum traction strength (MPa)	35
Maximum yield at 5 (%)	11
Thermal expansion (μstrain/°C)	77.39
Thermal conductivity (W/m °C)	0.26
Specific Heat (J/kg °C)	1575

**Table 4:** Information about the temperature ranges, the cooling fluid flow constants, and their ratios of improvement of the initial insert designs (without cooling channels) and the redesigns (with cooling channels).

Temperature and Fluid Parameters	Design without Cooling Channels	Design with Cooling Channels	Ratios of Improvement
Product temperature range (°C)	47–48	43.5–44.5	−7.4%
Insert core temperature range (°C)	45–47	40–45	−7.6%
Cooling fluid flow velocity (m/s)	N/A	3	(considered as turbulent flow)
Cooling fluid flow Reynolds number	N/A	5800	

**Table 5:** Information about operation times and their ratios of improvement of the initial inserts design (without cooling channels) and the re-design (with cooling channels).

Operation Times	Design without Cooling Channels	Design with Cooling Channels	Ratios of Improvement
Filling (s)	1	1	-
Holding (s)	8	8	-
Cooling (s)	50	46	−8%
Mould opening and ejector forward (s)	5	5	-
Total injection moulding cycle (s)	64	60	−6.25%

**Table 6:** Main parameters for the unit cost comparison for the scenarios of the initial insert designs (without cooling channels) and the redesigns (with cooling channels).

Scenario	Batch Sizes	Preparation Costs	Annual Costs of Equipment and Tooling (Excl. Machinery)	Processing Time per Part (Cycle Time)	Hourly Running Facility Costs	Processing Cost per Part	Other Costs per Unit Independent from the Batch Size (Materials)
	*B* (ut)	*Cprep* (EUR/year)	*Ci* (EUR)	*Tc* (s)	*Ch* (EIR/h)	*Cproc* (EUR/ut)	*Co* (EUR/ut)
(0) Original inserts	2,376,000	45	4500	8.0	21.09	0.047	0.05
(1) Inserts redesign	2,534,400	45	9600	7.5	21.09	0.044	0.05

**Table 7:** Main parameters for the unit cost comparison for the scenario based on the redesign of a full mould with conformal cooling in most surfaces (not only in the inserts).

Scenario	Batch Size	Preparation Costs	Annual Costs of Equipment and Tooling (Excl. Machinery)	Processing Time per Part (Cycle Time)	Hourly Running Facility Costs	Processing Cost per Part	Other Costs per Unit Independent from the Batch Size (Materials)
	*B* (ut)	*Cprep* (EUR/year)	*Ci* (EUR)	*Tc* (s)	*Ch* (EUR/h)	*Cproc* (EUR/ut)	*Co* (EUR/ut)
(2) Mould redesign	5,197,500	30	50,400	3.7	21.09	0.037	0.05

**Table 8:** Main parameters for the unit cost and production times comparison for the scenario based on the direct manufacturing of the swimming pool sink cage ribs via multi jet fusion.

Scenario	Batch Sizes	Preparation Costs	Annual Costs of Equipment and Tooling (Excl. Machinery)	Processing Time per Part (Cycle Time)	Hourly Running Facility Costs	Processing Cost per Part	Other Costs per Unit Independent from the Batch Size (Materials)
	*B* (ut)	*Cprep* (EUR/year)	*Ci* (EUR)	*Tc* (s)	*Ch* (EUR/h)	*Cproc* (EUR/ut)	*Co* (EUR/ut)
(3) Multi Jet Fusion	104	60,923.08	0	363.46	81.17	8.19	2
